# Effect of neurofeedback therapy on neurological post-COVID-19 complications (A pilot study)

**DOI:** 10.1371/journal.pone.0271350

**Published:** 2022-07-27

**Authors:** Mária Orendáčová, Eugen Kvašňák, Jana Vránová

**Affiliations:** Department of Medical Biophysics and Medical Informatics, Third Faculty of Medicine, Charles University in Prague, Prague, Czech Republic; Prince Sattam Bin Abdulaziz University, College of Applied Medical Sciences, SAUDI ARABIA

## Abstract

**Objective:**

Anxiety, fatigue and depression are common neurological manifestations after COVID-19. So far, post-COVID complications were treated by rehabilitation, oxygen therapy and immunotherapy. Effects of neurofeedback on post-COVID complications and their potential interrelatedness have not been studied yet. In this pilot study, we investigated the effectiveness of neurofeedback (Othmer method) for treatment of fatigue, anxiety, and depression after COVID-19.

**Methods:**

10 participants met inclusion criteria for having positive anamnesis of at least one of the following complications following COVID-19: fatigue, anxiety, and depression which were measured by questionnaires. ANOVA was used for calculating differences in questionnaire score before and after neurofeedback. Pearson’s correlation coefficient was used to calculate correlations between anxiety, depression and fatigue.

**Results:**

After five neurofeedback sessions, there came to significant reduction of severity of post-COVID anxiety and depression persisting for at least one month. Effect of neurofeedback on fatigue was insignificant. Severity of anxiety, fatigue and depression as well as reductions in depression and fatigue were positively correlated with each other.

**Conclusion:**

These findings showed effectiveness neurofeedback for reducing anxiety and depression after COVID-19 and for studying correlations between neurological complications after COVID-19. However, since our pilot clinical trial was open-label, it is hard to differentiate between neurofeedback-specific and unspecific effects on our participants. Future randomized controlled trials with more robust sample are necessary to investigate feasibility of neurofeedback for post-COVID neurological complications. The study has identification number trial ID ISRCTN49037874 in ISRCTN register of clinical trials (Retrospectively registered).

## 1 Introduction

Complications after COVID-19 occur in approximately 8–47.5% of COVID-19 survivors [1, 2]. Post-COVID-19 complications are defined as symptoms that occur or persist for at least 3 months after acute COVID-19 and that cannot be attributed to any other aetiology [3]. Neurological post-COVID-19 complications include manifestations such as fatigue, insomnia, headache, anxiety, depression, dizziness, and epileptic seizures [4–7]; many of these disorders occur in neurological conditions other than post-COVID-19 [8–11]. Post-COVID-19 complications have been already treated with immunotherapy [12, 13], oxygen therapy [14], pharmacological treatment [6] and rehabilitation [15]. The potential therapeutic benefit of non-invasive brain stimulation has already been considered as a promising treatment for COVID-19-related neurological complications [16]. To date, transcranial direct current stimulation has been found to reduce subjective fatigue in COVID-19 survivors [17].

Biofeedback (BFB) is a type of neuro-modulation method that is based on providing auditory and/or visual feedback to the participant based on changes in the activity of the participant’s selected biosignal modality [18–20]. Modalities of biofeedback reward include changes in brain activity dependent on blood oxygen content, heart rate [21, 22], and electroencephalographic (EEG) changes in brain activity [23]. EEG-based BFB is also referred to as neurofeedback (NFB). NFB is based on the principle of rewarding increases or decreases of the amplitude or coherence of selected EEG activity [18–20]. The EEG signal is sensed from electrodes placed on the participants’ head. Once the amplitude or coherence value of the selected EEG activity rewarded by the NFB reaches a value that is either equal to or greater than the reward threshold value, the participant receives visual and/or auditory feedback from the NFB device [18–20]. As a result, the brain associates the visual and auditory feedback of the NFB with the brain state rewarded by the NFB [18–20]. The clinical potential of NFB has already been documented in a number of psychiatric and neurological conditions such as anxiety [24], headaches [25], fatigue [26], sleep disturbances [27], cognitive functions [28, 29], and others [30, 31]. Because many of the above clinical conditions overlap to a huge extent with neurological symptoms after COVID-19, we hypothesized that NFB could improve neurological complications after COVID-19.

In this pilot study, we decided to investigate the effect of neurofeedback therapy on the following selected neurological complications after COVID-19: fatigue, anxiety and depression. Electrode locations used for NFB included the right and left temporal lobes (T3 and T4 according to the 10–20 system). The temporal lobes were chosen for the following reasons: first, abnormalities in the structure and/or activity levels in these brain regions in COVID-19 survivors who suffered complications after COVID-19 [32–35] and functional abnormalities in the temporal lobes after COVID-19 have been associated with some complications after COVID-19 [36, 37]. The temporal lobes are strongly involved in the central autonomic nervous system (CANS) [38] and the limbic system [39], both of which play a very important role in the regulation of emotion and autonomic functions [16, 39]. Therefore, we hypothesized that training the NFB of these regions could improve conditions such as anxiety, depression and fatigue, as these conditions may stem from dysfunction of the CANS and limbic system [40–42]. Second, abnormalities in brain metabolism in the temporal lobes have been associated with conditions such as anxiety [43, 44], depression [45, 46], fatigue [47, 48]. Third, the interhemispheric NFB protocol was chosen to balance right and left hemisphere activation. An imbalance between the level of left and right hemisphere activation can eventually lead to undesirable effects such as left-sided or right-sided headaches [87]. The rewarded individual frequency was individualized according to so-called Othmer method. Othmer method is based on finding and training the so-called individual optimal EEG frequency (IOF) [49–51]. The IOF is the width of the EEG frequency band that is associated with the subjective feeling of mental alertness, relaxation and a sense of calm, which are the subjective hallmarks of optimal arousal. On the other hand, too high EEG frequency rewarded by NFB can lead to symptoms of high arousal such as anxiety, insomnia, headaches [49–51]. Conversely, assuming that too low EEG frequency of NFB is rewarded, symptoms such as apathy, sadness, depressive feelings, and fatigue may occur, illustrating the prevalence of low arousal [49–51]. Given that some post-COVID-19 symptoms, such as anxiety, resemble high arousal symptoms, whereas other symptoms, such as depression and fatigue, may resemble low arousal, we hypothesize that Othmer NFB method could be beneficial in reducing the severity of COVID-19 due to normalization of arousal. In addition, NFB could also be beneficial for investigating whether there are correlations between the particular symptoms, which may be revealed by the correlated improvement in some specific symptoms following COVID-19. In particular, we hypothesize that IOF-NFB training will result in inter-correlated improvements of fatigue, depression, and anxiety. We hypothesize that inter-correlated improvements in anxiety, fatigue, and depression could occur due to the possible optimization of arousal by IOF and also due to the documented positive correlations between fatigue, anxiety, and depression in post-COVID condition [15, 52] as well as neuropathological conditions other than the post-COVID-19 condition [9, 10]. In this pilot study, we decided to investigate the effectiveness of neurofeedback for treatment of fatigue, anxiety and depression after COVID-19. Our aim of this study was to investigate whether the following hypotheses are true or false:

1. 5 sessions of NFB will significantly reduce fatigue, anxiety, and depression after COVID-19.
2. NFB-induced significant improvement in the above post-COVID symptoms will be still evident one week after NFB.
3. NFB-induced significant improvement in the above post-COVID symptoms will persist for one month after NFB.
4. Severity of fatigue, anxiety and depression will be positively correlated with each other.
5. mprovements in fatigue, anxiety and depression will be positively correlated with each other.

## 2 Methods

### 2.1 Participants

The following criteria were required for inclusion in this study: age at least 18 years (both males and females were enrolled), a positive history of Sars-CoV-2 infection confirmed by a positive antigen/RT-PCR/antibody test, and at least one of the following symptoms: fatigue, depression, and anxiety that were not present prior to Sars-CoV-2 infection. The specific symptoms should have been present or persisted for at least 3 months after confirmed Sars-CoV-2 infection and should not have been attributable to any other neurological disease prior to COVID-19. Participants should have been free of health problems prior to Sars-CoV-2 infection and medication-free or medication-stabilized in dose and type of drug for at least 3 months prior to the beginning of the study. As this is a pilot study, no specific age group was required.

**Fig 1:**
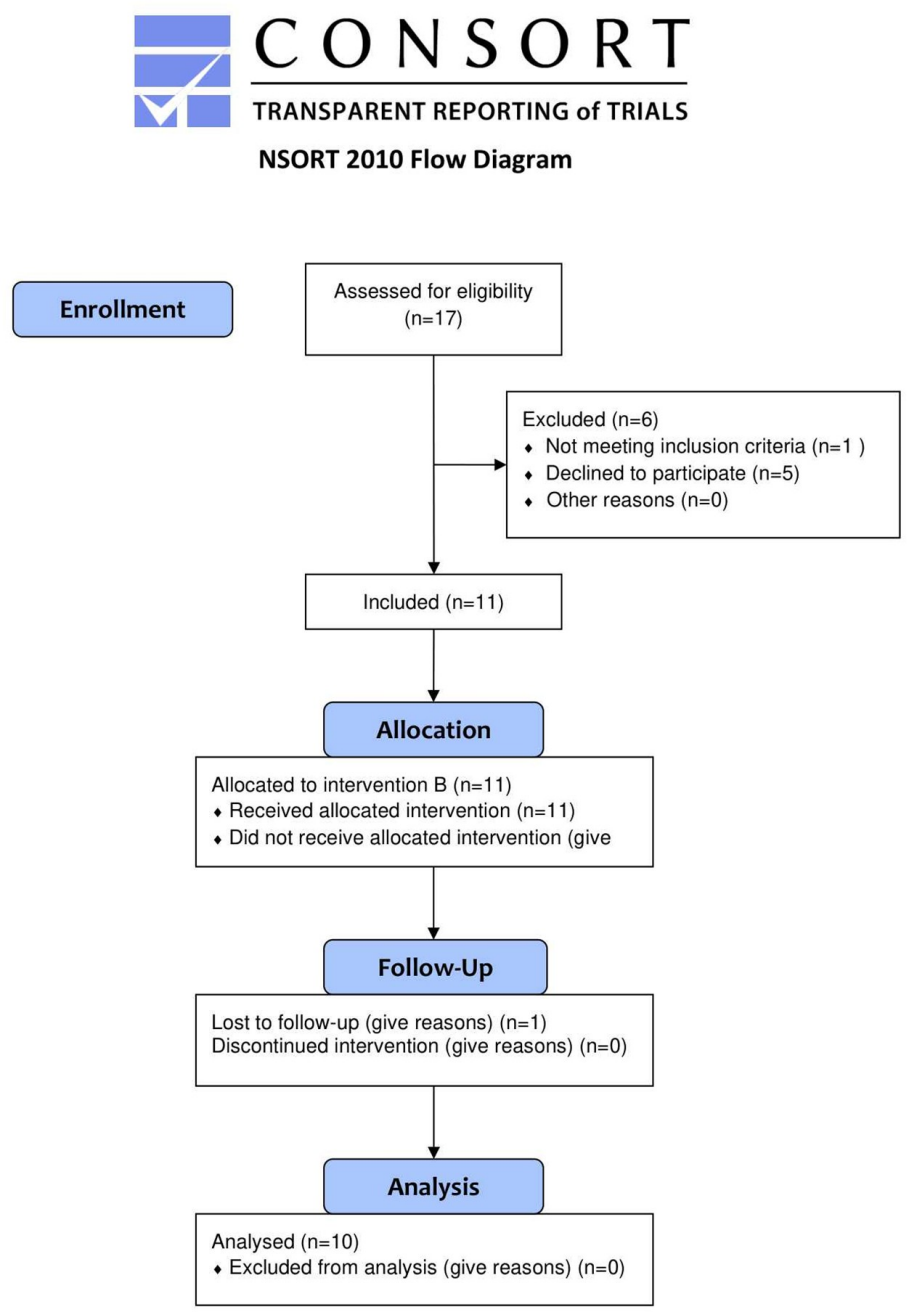
Flow diagram of recruitment of the participants.

In total, 17 participants were enrolled in the study. 6 of them did not meet inclusion criteria and/or they decided not to participate in the experiment. 1 participant has completed 4 NFB sessions and she dropped the experiment due to medical reasons. In total, 10 participants (3 males and 7 females) met inclusion criteria and completed all 5 NFB sessions and fill medical questionnaires and therefore they were included in the statistical analysis. Fig 1 shows flow diagram of enrolment of the participants.

### 2.2 Procedure

Participants have been invited to participate in this study, provided that they met inclusion criteria. Recruitment of the participants started on 2021-07-16 and ended on 2021-11-02. Participants were recruited online among COVID-19 survivors by means of online posting of recruitment leaflets in student facebook groups for students from Charles University in Prague, Czechia as well as in facebook online groups dedicated to people suffering from self-referred post-COVID complications. Experiments took place in Department of Medical Biophysics and Medical Informatics in Charles University in Prague, Czechia. Medical questionnaires were filled and submitted online 1–3 days prior to the first experimental session, then immediately after finishing all 5 NFB sessions, then one week after the termination of the experiment and finally month after termination of NFB experiment. Follow-up (observational) period ranged from 2021-08-11 and ended on 2021-12-22. The study was conducted in within-subject design. Pre-NFB and post-NFB data were compared within the same participant. In the first NFB session, informed consent has been signed. Then the short anamnestic semi-structured interview, related to details of dynamics of acute COVID-19 and post-COVID-19 symptoms, was given. Then the participants got familiarized with the NFB procedure. None of the participants had undergone NFB therapy prior to this experiment. The study has identification number trial ID ISRCTN49037874 in ISRCTN register of clinical trials (https://www.isrctn.com/ISRCTN49037874). The study has been approved by Ethic Committee of Third Faculty of Medicine, Charles University in Prague, Czechia and was conducted in accordance with the Code of Ethics of the World Medical. Association (Declaration of Helsinki for Human Experiments). The reason for retrospective registration of the study to clinical trial register is that at the time of approval of the study by Ethic Committee, the study was not considered as clinical trial. The authors confirm that all ongoing and related trials for this drug/intervention are registered. All participants signed and submitted informed consent (written form) during the first session prior to the whole experimental procedure. Protocol of the study (both English and original Czech version) as well as estimated time schedule for data collection are included in S1 Protocol.

### 2.3 Neurofeedback

NFB training included five NFB sessions which were completed within two weeks. Time period of one NFB training session was individualized for each participant and ranged between 25–45 minutes. The reason for this time variability stemmed from the process of finding IOF, as there was the effort to end each NFB session with established NFB-rewarded EEG frequency to be as optimal as possible. Two NFB sessions were supposed to be completed in the first week and three sessions were supposed to be completed in the second week or vice versa. The time of the beginning of NFB session was individualized with regard to personal time capacities of each participants but it was supposed to be kept fixed for all 5 NFB sessions with tolerable deviation +/- 3 hours. This was done in order to minimize possible effects of infradian rhythms on NFB training. NFB-rewarded EEG frequency bandwidth was individualized for all participants according to Othmer method. The first experimental session involved finding of IOF. Participants were aware of receiving NFB intervention. They were instructed to sit comfortably and adjust their mental activity to the mental state which is associated with the presence of visual and auditory feedbacks from NFB device. In accordance with Othmer method; we started with NFB up-regulation of 12–15 Hz EEG activity for 2 minutes. After finishing, participants were asked questions related to their subjective feeling, such as: Did you feel comfortable during the training? Was there any headache, tingling or feeling of tension? Provided that the participants stated feelings such as anxiety, body tension and headache, NFB-rewarded EEG frequency was lowered 1 Hz. On the other hand, provided that the feelings of apathy, somnolence or sadness were reported, NFB-rewarded frequency was increased 1 Hz. IOF was adjusted for each 5 NFB sessions based on intra-session and inter-session reports of participants. To investigate inter-session effects of NFB-rewarded frequencies, the participants were asked the following questions: Did you have any headaches, anxiety, dizziness or nightmares? How was your sleep? How was your mood? Did you have some inexplicable feelings of apathy or sadness? Have you been more fatigued or somnolent as usual? Provided that there was occurrence of symptoms such as onset insomnia, nightmares, dizziness, headaches and anxiety, NFB-rewarded frequency was lowered 1 Hz. On the other hand, provided that there were reports of inter-session fatigue, somnolence, sleepiness, sadness, NFB-rewarded frequency was increased 1 Hz. In order to be NFB-rewarded frequency considered IOF, it had to meet the following two criteria: First, in intra-session period, NFB-rewarded frequency had to be associated with subjective feelings of relaxation, calmness or at least with euthymic state. At the same time, any of aforementioned symptoms indicating either high or low arousal were not supposed to be present. Second, in relation to inter-session period, at least one positive outcome, such as subjective improvement in sleep, mood etc., was supposed to be present. Two active electrodes were placed at right and left temporal areas (T3-T4, 10–20 system). Ground electrode was put at right earlobe. Electrodes were filled with Ten 20 Past (Neurodiagnostic Electrode Paste, Weaver and Company, made in USA) used for making electrodes to be fixed at the scalps of participants. Conductive electrode gel was used to keep impedance as low as possible (Sigma gel, Parker laboratories, Fairfield, made in New Jersey, USA). NFB feedbacks included visual as well as auditory modalities.

**Fig 2:**
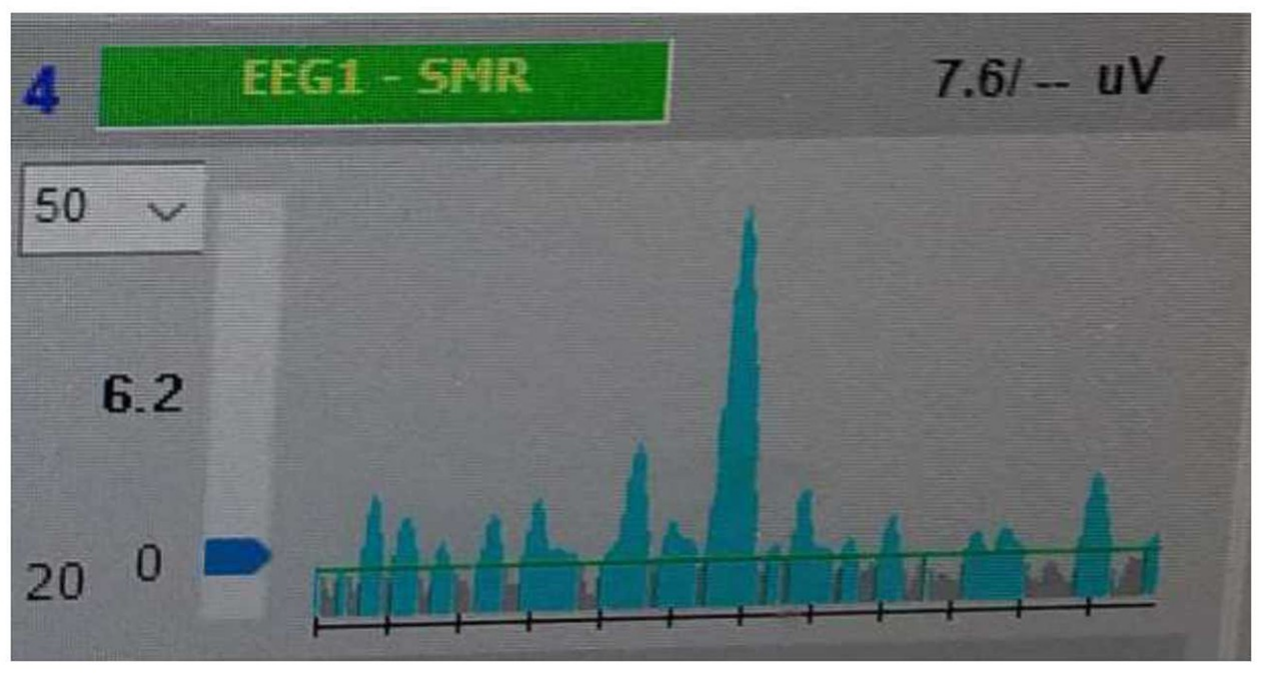
Principle of visual NFB feedback. Figure 2 depicts the underlying principle of NFB rewarding visual feedbacks. When NFB-rewarded selected EEG activity kept below reward threshold, online EEG graph was grey. As soon as amplitude of EEG activity was at least as high as reward threshold value, EEG graph got blue. Horizontal green line depicts reward threshold. Black line divided into 12 segments (each segment represents one second) (axis X) depicts time window. Axis y represents amplitude of EEG in pV (microvolt). Blue arrow in Y axis serves for manual adjustment of NFB reward threshold. 6.2 values next to axis Y (left side) indicates minimal value in pV which is necessary to be reached to get NFB feedback (blue EEG activity). 7.6 1.1V (the upper right quadrant indicates the average reward value within the current time window. 4 EEG1 -SMR is the title of this target online EEG graph predefined by NFB software Deymed Diagnostic (version 11).

For visual feedbacks, watching the computer screen was required. On the computer screen, there was graph of ongoing EEG activity of the underlying NFB-rewarded bandwidth. When NFB-rewarded selected EEG activity kept below reward threshold, online EEG graph was grey. As soon as amplitude of EEG activity was at least as high as reward threshold value, EEG graph got blue. Reward threshold was adjusted manually by certified neurofeedback therapist to keep frequency of feedbacks as constant as possible. There was the effort to adjust reward threshold to ensure both ‘‘grey” intervals of no feedbacks and ‘‘blue” intervals associated with NFB-feedbacks are present in order to make brain able to differentiate between the states of being and states of not being rewarded by NFB device. Fig 2 depicts the principles of visual NFB feedback.

For auditory feedback, the participants were given ‘‘Quiet Bong” sound which was selected from sound options of our NFB software (Deymed Diagnostics, version 11).

### 2.4 Questionnaires

For indicating the severity of pre-NFB and post-NFB post-COVID-19 fatigue, anxiety and depression, the following standardized medical questionnaires were used: Fatigue Assessment Scale for measuring the level of fatigue, Beck Anxiety Inventory for measuring the level of anxiety and Beck Depression Inventory (version 2) for measuring the level of depression.

#### 2.4.1 Fatigue Assessment Scale

For measuring the level of fatigue, Fatigue Assessment Scale was used. Fatigue Assessment Scale includes 10 questions related to the subjectively perceived level of fatigue. For each of these 10 questions, there exist 5 possible answers from which just one is supposed to be chosen. These 5 answers are the following: never, rarely, sometimes, often and always. Answer “never” is scored by 1 point, answer ‘‘rarely” is scored by 2 points, answer ‘‘sometimes” is scored by 3 points, the answer ‘‘often” is scored by 4 points and the answer ‘‘always” is scored by 5 points. This scoring algorithm is applied for the questions, 1,2,3,5,6,7,8 and 9. For the questions 4 and 10, inverse scoring of the answer is used. Score less than 22 indicate no fatigue; score higher than 22 indicate fatigue [53]. For being included in this study in the subgroup of participants analyzed for post-COVID-19 fatigue (Section 3.5), score of at least 22 points was required.

#### 2.4.2 Beck Anxiety Inventory

For measuring the level of anxiety, Beck Anxiety Inventory was used. Beck Anxiety Inventory includes 20 questions regarding the severity of occurrence of a various anxiety symptom within the period of the last month. For each question, there exist 4 possible answers: The first answer is scored by 0 points and states that particular anxiety symptom is not present. The second possible answer is scored by 1 point and states that the particular anxiety symptom was mildly unpleasant. The third possible answer is scored by 2 points and states that the particular anxiety symptom was moderately unpleasant. The third possible answer is scored by 3 points and states that the particular anxiety symptom was perceived as a severe and very disturbing. For indicating the level of anxiety, total score has been calculated for all 20 questions. 0–21 points indicate low anxiety level. 22–35 points indicate moderate anxiety level. Score higher than 36 points indicate high level of anxiety [88]. In order to be included in the subgroup of participants suffering from post-COVID-19 anxiety (Section 3.6), the score at least 22 points (moderate level of anxiety) was used as cut-off.

#### 2.4.3 Beck Depression Inventory

To measure the level of depression, Beck Depression Inventory (Second version) was used. Beck Depression Inventory includes 21 questions. For each question, there exist 4 possible answers from which one answer is supposed to be chosen. The answers are scored by 0–4 points. Total score obtained from answering all 21 questions indicates the level of depression. 0–10 points indicate normal state. 11–16 score indicate mild mood disturbance. 17–30 points indicate borderline clinical depression. 20–30 points indicate moderate depression. 31–40 points indicate severe depression and score higher than 40 points indicate extreme depression [54]. To be involved in this study in the subgroup of participants analyzed for post-COVID-19 depression (Section 2.4), the score at least for borderline clinical depression (17 points) was used as cut-off.

### 2.5 Statistical analysis

The study was conducted in within-subject (repeated measures) design. The score of Fatigue Assessment Scale, Beck Depression Inventory and Beck Anxiety Inventory was calculated. As all these observed quantitative variables fulfil the normal distribution conditions as revealed by Kolmogovov-Smirnov test, the means and standard deviations were presented. For comparison among pre-NFB and post-NFB data (data immediately, one week and one month after NFB) the repeated measures ANOVA with multiple comparisons with Bonferroni test was used. To find the relationship between fatigue, anxiety and depression, Pearson’s correlation coefficients were calculated. Pearson’s correlation coefficients were calculated also to find the associations between improvement of scores for fatigue, depression and anxiety measured by Fatigue Assessment Scale, Beck Anxiety Inventory and Beck Depression Inventory. Descriptive statistical values such as mean value, minimum, maximum and standard deviation are presented in Table 1 in S1 File. Due to relatively small sample size of our participants and imbalance in distribution between men and women (3 males and 7 females), age and sex-dependent possible differences were not calculated.

For statistical analysis STATISTICA version 14.0.0.15 (TIBCO Software Inc.) was used. The required sample size for all statistically significant correlation coefficients was calculated using G*Power software version 3.1. All tests were considered to be statistically significant at the level of p<0.05.

## 3 Results

### 3.1 Characteristics of participants

Age of the participants ranged from 19 to 46 years (median = 21). 8 participants had positive confirmation by Sars-CoV-2 infection done by nasopharyngeal RT-PCR test. 1 participant had positive nasopharyngeal antigen test and since he was symptomatic at the time of being positively tested (chest pain, sore throat, headaches and fatigue) he went into quarantine without the confirmation RT-PCR test. 1 participant was positively tested for IgG antibodies done by ELISA method. The participant decided to take IgG testes due to be suspected to recover from symptomatic COVID-19 after epidemiological relevant contact with person with confirmed Sars-CoV-2 infection. In relation to acute COVID-19 symptoms (and suspected COVID-19 in case of 1 participant with positive IgG test), distribution of acute COVID-19 symptoms was as follows: headaches (70% of participants), fatigue (80% of participants), elevated temperature (30% of participants), fever (20% of participants), dyspnoea (20% of participants), chest pain (20% of participants), olfactory impairment (10% of participants), gustatory impairment (30% of participants), cough (30% of participants) and sore throat (30% of participants). None of the participants was hospitalized due to COVID-19. In relation to the participants having positive nasopharyngeal swab confirming Sars-CoV-2 infection, the median period between their acute Sars-CoV-2 infection and enrolment into our study due to persisting post-COVID-19 problems, ranged from 3–19 months (median = 12 months). The participant, who was tested positive for IgG antibody, was enrolled in our study 6 months after her positive IgG test due to persisting neurological symptoms. In relation to fatigue, depression and anxiety, which were target post-COVID-19 complications of our interest, there was the following distribution within the participants: fatigue (90% of participants), anxiety (80% of participants) and depression (60% of participants). In relation to observed post-COVID-19 complications of our interest, according to semi-structured entering interview as well as data from standardized medical questionnaires (Fatigue Assessment Scale for measuring level of fatigue, Beck Anxiety Inventory for measuring the level of anxiety and Beck Depression Inventory) for measuring the level of depression, there was the occurrence of single symptom in 1 participant, occurrence of 2 simultaneous symptoms in 3 participants, and occurrence of 3 simultaneous symptoms in 6 participants.

### 3.2 Fatigue

According to data from Fatigue Assessment Scale, 9 from 10 participants (N = 9) met inclusion criteria for fatigue (minimum: 22 points, maximum: 45 points, mean: 32.77778 points, S.D.:9.06612). In comparison to pre-NFB data from Fatigue Assessment Scale, there came to insignificant reduction of score of Fatigue Assessment Scale immediately after 5 NFB sessions (p = 0.083583). Also, relative to pre-NFB data, insignificant reduction of score of Fatigue Assessment Scale was revealed 1 week after termination of NFB (p = 0.122952) as well as 1 month after termination of NFB (p = 0.088454). Raw data, used for this analysis, are included in Table 2 in S1 File. Table 1 demonstrates our findings.

**Table 1:** Score for fatigue before and after NFB measured by Fatigue Assessment Scale.

	LSD Test; Variable: Fatigue Assessment Scale. Marked differences are significant p<0.050000			
Condition of measurement	(1) Before NFB	(2) Immediately after NFB	(3) One week after NFB	(4) One month after NFB
**Results**	N = 9	N = 9	N = 9	N = 9
		M = 25.889	M = 26.667	M = 26.000
	M = 32.778	P = 0.083583	P = 0.122952	P = 0.088454

The following table depicts p values for difference between pre-to-post NFB data for score for Fatigue Assessment Scale. Abbreviation LSD stands for Least Significance Difference. M stands for mean value. N indicates number of tested participants for the particular variable. P stands for p value. Numbers in the brackets mean the following: (1) means data before NFB, (2) means data immediately after NFB, (3) means data 1 week after NFB and (4) means data after 1 month after NFB.

### 3.3 Anxiety

According to data from Beck Anxiety Inventory 8 from 10 participants (N = 8) met inclusion criteria for anxiety (at least moderate level of anxiety) (minimum: 25 points, maximum: 49 points, mean: 32.50000 points, S.D.: 8.94427). In comparison to pre-NFB data from Beck Anxiety Inventory, there came to significant reduction of score of Beck Anxiety Inventory 1 week after 5 NFB sessions (p = 0. 014910) as well as 1 month after termination of NFB (0.0088334). Beck Anxiety Inventory was not administrated to participants immediately after termination of the last (5^th^) NFB session due to the characteristics of that particular questionnaire-assessment of 2 weeks is too short period for Beck Anxiety Inventory [88]. Raw data, used for this analysis, are included in Table 3 in S1 File. Table 2 shows our findings.

**Table 2:** Score for anxiety before and after NFB measured by Beck Anxiety Inventory.

	LSD Test; Variable: Beck Anxiety Inventory. Marked differences are significant p<0.050000			
Condition of measurement	(1) Before NFB	(2) Immediately after NFB	(3) 1 week after NFB	(4) 1 month after NFB
	M = 32.500	M = 0.0000	M = 19.750	M = 18.500
**Results**	N = 8	Not measured	N = 8	N = 8
	M = 32.500		M = 19.750	M = 18.500
			P = 0.014910	P = 0.008334

The following table depicts p values for difference between pre-to-post NFB data for score for Beck Anxiety Inventory. Abbreviation LSD stands for Least Significance Difference. M stands for mean value. N indicates number of tested participants for the particular variable. P stands for p value. Numbers in the brackets mean the following: (1) means data before NFB, (2) means data immediately after NFB, (3) means data 1 week after NFB and (4) means data after 1 month after NFB.

### 3.4 Depression

According to data from Beck Anxiety Depression Inventory (2.version) 6 from 10 participants (N = 6) met inclusion criteria for suffering from depression (at least borderline level of depression) (minimum: 17 points, maximum: 42 points, mean: 26.66667 points, S.D.: 9.28901). In comparison to pre-NFB data from Beck Depression Inventory, there came to significant reduction of score of Beck Depression Inventory immediately after 5 NFB sessions (p = 0.018763). Also, relative to pre-NFB data, significant reduction of score of Beck Depression Inventory was present 1 week after termination of NFB (p = 0.018763) as well as 1 month after termination of NFB (0.027231). Raw data, used for this analysis, are included in Table 4 in S1 File. Table 3 shows our findings.

**Table 3:** Score for depression before and after NFB measured by Beck Depression Inventory.

	LSD Test; Variable: Beck Depression Inventory. Marked differences are significant p<0.050000			
Condition of measurement	(1) Before NFB	(2) Immediately after NFB	(3) 1 week after NFB	(4) 1 month after NFB
**Results**	N = 6	N = 6	N = 6	N = 6
	M = 26.667	M = 14.500	M = 14.500	M = 15.333
		P = 0.018763	P = 0.018763	P = 0.027231

The following table depicts p values for difference between pre-to-post NFB data for score for Beck Depression Inventory. Abbreviation LSD stands for Least Significance Difference. M stands for mean value. N indicates number of tested participants for the particular variable. P stands for p value. Numbers in the brackets mean the following: (1) means data before NFB, (2) means data immediately after NFB, (3) means data 1 week after NFB and (4) means data after 1 month after NFB.

### 3.5 Correlations between fatigue, anxiety and depression measured by Fatigue Assessment Scale, Beck Depression Inventory and Beck Anxiety Inventory

In this analysis, data of all 10 participants (N = 10) were calculated. Raw data, used for this analysis, are included in Table 5 in S1 File. In relation to pre-NFB data, there was revealed a significant positive correlation between the level of fatigue and level of depression (p = 0.001). This correlation between depression and fatigue remained significant even immediately after termination of NFB (p = 0.005), 1 week after NFB (p = 0.006) and 1 month after termination of NFB (p = 0.002). Data obtained from questionnaires 1 week after termination of NFB revealed significant positive correlation between fatigue and anxiety (p = 0.003) and somewhat weaker but still significant positive correlation between anxiety and depression (p = 0.018). Both of these positive correlations remained still significant 1 month after termination of NFB. (p = 0.005 for positive correlation between anxiety and fatigue and p = 0.004 for positive correlation between depression and anxiety). Other correlations between the particular analyzed variables were insignificant (Table 4).

**Table 4:** Correlations between fatigue, anxiety and depression measured by Fatigue Assessment Scale, Beck Depression Inventory and Beck Anxiety Inventory.

Variable	Sample (N)	Correlation Coefficient (R)	P value (P)
**Correlation between fatigue and anxiety before NFB**	N = 10	R = 0.4892	P = 0.51
**Correlation between fatigue and depression before NFB**	N = 10	R = 0.8619	P = 0.001
**Correlation between anxiety and depression before NFB**	N = 10	R = 0.4916	P = 0.149
**Correlation between fatigue and anxiety immediately after NFB**	/	/	/
**Correlation between fatigue and depression immediately after NFB**	N = 10	R = 0.4916	P = 0.005
**Correlation between anxiety and depression immediately after NFB**	/	/	/
**Correlation between fatigue and anxiety one week after NFB**	N = 10	R = 0.8346	P = 0.003
**Correlation between fatigue and depression one week after NFB**	N = 10	R = 0.7990	P = 0.006
**Correlation between anxiety and depression one week after NFB**	N = 10	R = 0.7218	P = 0.018
**Correlation between fatigue and anxiety one month after NFB**	N = 10	R = 0.8065	P = 0.005
**Correlation between fatigue and depression one month after NFB**	N = 10	R = 0.8542	P = 0.002
**Correlation between anxiety and depression one month after NFB**	N = 10	R = 0.3512	P = 0.004

The following table depicts p values for correlations between fatigue, anxiety, and depression before, immediately, 1 week and 1 month after NFB. Abbreviation N stands for number of participants used for the particular analysis. Abbreviation R stands for Correlation Coefficient. Abbreviation P indicates value. The symbol ,,/,, indicates no measurement was done.

Graphical demonstrations of positive correlations between fatigue, depression and anxiety before, immediately, one week and one month after NFB can be seen in Figs 1–10 in S1 File.

### 3.6 Correlated reduction of score of Fatigue Assessment Scale, Beck Anxiety Inventory and Beck Depression Inventory

In this analysis, data of all 10 participants (N = 10) were calculated. Raw data, used for this analysis, are included in Table 5 in S1 File. Our analysis revealed significant positive correlation between pre-to-immediately post-NFB reduction of score for Fatigue Assessment Scale and score for Beck Depression Inventory (p = 0.028). Positive correlation between reduction of score for Fatigue Assessment Scale and score for Beck Depression Inventory persisted 1 week after termination of NFB (pre-NFB data vs. data 1 week after NFB, p = 0.001) as well as 1 month after termination of NFB (pre-NFB data vs. data 1 month after NFB, p = 0.031). Correlations between other variables were insignificant (Table 5).

**Table 5:** Correlated reductions of score of Fatigue Assessment Scale, Beck Anxiety Inventory and Beck Depression Inventory.

Variable	Sample (N)	Correlation Coefficient(R)	P value (P)
**Correlated reductions between anxiety and fatigue (before vs. immediately after NFB)**	/	/	/
**Correlated reductions between fatigue and depression (before vs. immediately after NFB)**	N = 10	R = 0.6862	P = 0.028
**Correlated reductions between anxiety and depression (before vs. immediately after NFB)**	/	/	/
**Correlated reductions between anxiety and fatigue (before vs. one week after NFB)**	N = 10	R = -0.1986	P = 0.582
**Correlated reductions between fatigue and depression (before vs. one week after NFB)**	N = 10	R = 0.8862	P = 0.001
**Correlated reductions between anxiety and depression (before vs. one week after NFB)**	N = 10	R = -0.0773	P = 0.832
**Correlated reductions between anxiety and fatigue (before vs. one month after NFB)**	N = 10	R = -0.1515	P = 0.676
**Correlated reductions between anxiety and depression (before vs. one month after NFB)**	N = 10	R = 0.3512	P = 0.324

The following table depicts p values for cross-correlated improvements between fatigue, anxiety, and depression before, immediately, 1 week and 1 month after NFB. Abbreviation N stands for number of participants used for the particular analysis. Abbreviation R stands for Correlation Coefficient. Abbreviation P indicates value. The symbol ,,/,, indicates no measurement was done.

Graphical demonstrations of positive correlations between reductions in fatigue, depression and anxiety before, immediately, one week and one month after NFB can be seen in Fig 11–17 in S1 File.

## 4 Discussion

In accordance with our criteria of IOF (Section: 3.6 Neurofeedback) for all participants, target EEG NFB-rewarded frequency has been individualized and adjusted within 5 NFB sessions with regard to the particular participant. Regarding intra-session experimental period potential unwanted side-effects of NFB revealed via subjective reports of increased fatigue, and/or muscle tension and/or occurrence of headaches were temporarily present as follows: in all 10 participants in the first experimental session, in the second NFB session in 5 participants, in the third NFB session in 2 participants and in fourth and fifth session for 1 participant. Decreasing number of participants experiencing these unwanted symptoms with progressing session number is likely to speak in favour of the effect of adjustment of NFB-rewarded EEG frequency as aforementioned unwanted symptoms were associated only with NFB training of some particular EEG frequencies whereas during training of other EEG frequencies, such unwanted symptoms were not present. Based on subjective reports of the participants, if these unwanted symptoms occurred, they did not have a longer persistence than for several hours. However, in order to be able to differentiate between NFB-related specific effects, nocebo effect and others, inclusion of control group receiving sham NFB-related feedback as well as of control group receiving no NFB-related feedback should be included.

Based on Beck Depression Inventory (version 2), 5 NFB sessions completed within 2 weeks resulted in a significant reduction in depression immediately after NFB, Such reduction of depressive symptoms persisted one week as well as one month after termination of NFB. Our results are in line with the outcomes of other studies reporting reduction in levels of depression after NFB [55–59]. There are some novel findings being brought by our study related to the effects of NFB on post-COVID depression: First, to the best of our knowledge, Othmer method of training OIF in temporal lobes has not been yet investigated regarding its effects on depression. So far, a various biofeedback modalities have been scrutinized in relation to their effects of depression such as quantitative electroencephalogram- based biofeedback (QEEG-based NFB) [58], functional magnetic resonance based neurofeedback (fMRI-based NFB) [56, 59], infralow NFB [60], alpha asymmetry NFB training [57] peak alpha frequency NFB training [55] and heart rate-based biofeedback (HRV-based BFB) [61]. Second, NFB as such has not been studied yet in the connection to post-COVID depression.

Compared to baseline condition (prior to NFB sessions), there was significant reduction of anxiety level one week as well as one month after completion of all 5 NFB sessions. In comparison to data one week after NFB (p = 0.014910), the levels of reduction of anxiety were significantly greater 1 month after NFB (p = 0.008334). Apart from possible improvement of anxiety caused by NFB, this trend might be also possibly attributable to natural time-dependent recovery from post-COVID condition as post-COVID anxiety was demonstrated to improve with time [62–64] though some studies did not prove such trend [65]. However, since we did not include control group in our study (sham NFB), it is difficult to differentiate between factors related to NFB-induced improvement and effects of time-dependent natural recovery processes, placebo effects and other. Relative to documented effects of NFB on anxiety, our results seem to be in line with other studies reporting reduction of anxiety after NFB intervention [57, 60, 66–68].

To the best of our knowledge, this is the first study demonstrating beneficial influence of Othmer method NFB in temporal lobes on anxiety. So far the following NFB modalities were used for the investigation of their effects on anxiety: fMRI-based NFB [69], infralow NFB [60], alpha asymmetry NFB training [57, 66] and HRV-based BFB [70], up-regulation of alpha and simultaneous down-regulation of beta EEG activity combined with alpha-theta training [68] and sensorimotor rhythm up-regulation [67]. Second, to the best of our knowledge, this is the first study investigating the effect of NFB as such on post-COVID anxiety. It remains questionable for how long NFB-associated improvement of post-COVID symptoms would persist and whether such period would be universal or unique per each particular post-COVID symptom. In our study, improvement of anxiety and depression was evident immediately after 5 sessions of NFB which is in accordance of other NFB studies finding improvement of anxiety after 5 NFB sessions [66]. Since we did not follow observation of our participants longer than 1 month after termination of NFB, it is questionable for how long period the improvements of post-COVID anxiety and depression persisted. Also, no sham NFB group was involved. For that reason, it cannot be differentiated whether long-lasting improvements in depression and anxiety are attributable to NFB, natural time-dependent recovery or other factors and for how long NFB-induced behavioral improvements may persist for. There are some evidences speaking in favour of achieving remission of some symptoms after 5 sessions of NFB [71]. This notion might have been at least partially mimicked in case study in which 5 tACS sessions led to long-lasting improvement of depression in major depressive disorder but there came to full relapse after 6 months after termination of tACS experiment [72]. Therefore, in relation to future research, we strongly propose including longer observation period to investigate dynamics of post-COVID manifestation for a long period after termination of therapeutic intervention.

In contrast to anxiety and depression, no significant pre-to-post NFB difference was observed for the levels of fatigue measured by Fatigue Assessment Scale. Although, there are some studies reporting significant reductions in fatigue following NFB, compared to our study, much greater number of NFB sessions was used in other studies ranging between 20–40 NFB sessions [26, 73–75]. Similar to our findings, 5 sessions of NFB combined with hypnosis treatment revealed mild reduction in fatigue reaching no statistical significance [76]. These outcomes might possibly speak in favour of necessity of inclusion of a greater number of NFB sessions to successfully improve post-COVID fatigue-related condition. In contrast to NFB, 4 sessions of transcranial direct current stimulation (tDCS) were showed to be effective at reducing subjective fatigue of post-COVID fatigue immediately after tDCS intervention [17]. These striking differences between effectiveness of tDCS and our NFB approach for overcoming post-COVID fatigue might indicate different levels of efficacy of these two neuromodulation methods and/or different effects of selection of different brain areas as in Workman et al (2021) study left motor cortex was selected while in other study, bilateral temporal lobes were chosen. In addition, 5 NFB sessions of IOF might be insufficient not only due to small number of NFB sessions as such but also due to the process of finding IOF. Since fatigue may occur as a result of unwanted side-effects of NFB [77] and in Othmer method the presence of fatigue may indicate non-optimal NFB-rewarded EEG frequency [50], it is possible that initial session/s of training of non-optimal EEG frequencies during the search for the IOF might have dampened possible beneficial effects of later found IOF on NFB-related alleviation of fatigue. However, in our study, no objective measurements were done to study structural and/or metabolic changes of brain activity prior, during and after NFB that would help to clarify this issue. Last but not least, it is necessary to take into consideration the fact that post-COVID fatigue is complex multi-faceted condition [78, 79] which may possibly stem from neural [79] but from extra-neural aetiologies as well l [79, 80], for example, from muscular dysfunction [79] and impairment of gut microbial in the intestine [80]. For that reason, it cannot be excluded that there was heterogeneity in the aetiology of post-COVID fatigue among our participants. NFB might not be equally effective treatment for all aetiologies of post-COVID fatigue, for instance, it might be less effective in treating post-COVID fatigue stemming from extra-neural sources than for the fatigue originating from neural causes. Nevertheless, no medical examination of the participants was done prior to NFB experiment to determine the exact aetiology of post-COVID fatigue.

Regarding the possible inter-relatedness of post-COVID fatigue, anxiety and depression, significant positive correlation between depression and fatigue has been found before as well as immediately after NFB intervention which persisted one week and one month after NFB. These findings seem to be in line with other study discovering positive correlation between post-COVID fatigue and depression [15]. and between fatigue and anhedonia in COVID-19 survivors [81]. These outcomes may speak in favour of common mechanisms responsible for exacerbation of post-COVID depression and fatigue and/or casual inter-relatedness between these manifestations. For instance, it is possible that depression may secondarily trigger fatigue or vice versa. Intriguingly, prior to NFB, there were no correlations between depression and anxiety, and between anxiety and fatigue. However, data from Beck Anxiety Inventory, Beck Depression Inventory and Fatigue Assessment Scale revealed significant positive correlation between anxiety and depression, and fatigue and anxiety one week as well as one month after termination of NFB. Positive correlation between post-COVID anxiety and fatigue is in agreement with the findings of the other study [52]. Also, positive correlation between post-COVID depression and anxiety was reported in another study [65]. Remarkably, in our study, there was absence of positive correlations between anxiety and fatigue, and anxiety and depression before NFB and consequent significant correlations between anxiety and fatigue, after NFB persisting for at least 1 month. These findings might suggest that NFB-related processes might have somehow induced inter-relatedness between fatigue, anxiety and depression, maybe possibly due to IOF-related modulation of arousal since in-optimal arousal is linked with fatigue, depression and anxiety [50, 57, 82]. However, no objective measurements of arousal were exploited in this study to reject or confirm this hypothesis. Also, control group (sham NFB) was not included to be able to differentiate whether the occurrence of positive correlations between fatigue and depression, and between depression and anxiety is exclusively associated with NFB or not.

Finally, we investigated whether there will be inter-correlated improvement of multiple post-COVID neurological symptoms after NFB. Score from Beck Depression Inventory and Fatigue Assessment Scale revealed significant inter-correlated improvement of fatigue and depression, which was present immediately, one week and one month after NFB. Our outcomes are in line with evidences that NFB can simultaneously improve fatigue, anxiety and depression [60]. Also, our findings seems to at least partially mimic the findings study done by Hayden et al, (2021) in which inter-correlated improvement of fatigue and depression was present after 3 weeks of pulmonary rehabilitation [15]. These outcomes might indicate possible inter-relatedness between post-COVID fatigue and depression as well as their common responsiveness to various kinds of therapies. So far, it is unknown whether interrelatedness between fatigue and depression and their common responsiveness to various kinds of therapies is specific to post-COVID condition or it is more universal. In patients suffering from multiple sclerosis, 5 sessions of tDCS led to simultaneous reduction of fatigue, pain and depression [9] possibly indicating some kind of universal inter-relatedness and common responsiveness of fatigue and depression to non-invasive brain modulation method, regardless the aetiology of these two conditions. However, future research should be done to investigate these issues.

Finally, it is necessary to mention potential limitations and strengths of Othmer method of training IOF as well as its documented feasibility in the treatment of other neurological conditions. So far, Othmer method of training IOF has been found to be effective in treating various conditions such as epileptic seizures [83], pain [84], impairment of executive functions, mood swings and other conditions related to instabilities in emotional regulation [85]. It can be seen these documented beneficial effects of Othmer method of finding IOF at least partially overlap with the findings of our study in which improvement of emotional problems such as anxiety and depression was seen in post-COVID participants. Based on these outcomes, we propose it might be interesting to conduct cross-sectional study focusing on comparison of effects of this kind of NFB modality on populations suffering from anxiety and depression having a different aetiology than post-COVID condition in order to learn whether responsiveness to Othmer method of finding IOF depends on aetiology of the particular clinical condition or not. Since it is unclear so far whether Othmer method of finding IOF would be equally effective in treating similar clinical conditions caused by different aetiologies, we believe that such kind of cross-sectional investigation might help to clarify this issue. So far, such type of cross-sectional study was done to compare effects of various NFB modalities, such as infra-low (ILF) NFB, classical frequency training, quantitative electroencephalogram-based (QEEG) NFB on pain, insomnia, anxiety, fatigue and depression on cancer patients vs. non-cancer ones. Although improvement of these symptoms was found in both populations, compared to cancer patients, significantly greater reduction of severity of these symptoms was present in non-cancer participants [86]. These findings showed there are differences of responsiveness to NFB between participants suffering from the same/very similar investigated clinical conditions caused by different etiologies. For that reason, it is possible such variability might be awaited in NFB effects between clinical symptoms caused by post-COVID condition and the same/very similar symptoms having different aetiology, however, as mentioned, the future study should shed more light on this issue. Last but not least, from longitudinal point of view, it is necessary to mention that Othmer method of finding IOF may be associated with session-to-session inconsistency in NFB protocols due to adjustment of rewarded EEG frequency bandwidth to find of rewarded EEG frequency that would meet criteria to be recognized as IOF [85]. Consequently, when comparing the effects of IOF protocol among the participants of the investigated population, there might be significant differences in effects of IOF training due to possible inter-individual and intra-individual differences in responses of the participants to that kind of treatment. We propose this limitation might be at least partially solved by administration of greater number of NFB session because of two reasons: First, to investigate whether IOF, once found, is stable during the long-term NFB period and whether its dynamics (stability vs. changes in time) is universal or varying regarding to the particular clinical symptoms caused by a different aetiology. Second, provided that IOF would be stable once found, application of greater number of NFB session (compared to only 5 sessions used in our study) would potentially eliminate variable effects of initial NFB sessions in which great variability of training NFB protocols is very likely to be present due to adjustment of training EEG frequency to meet criteria of being determined as IOF. Finally, it remains unknown whether other biofeedback modalities, such as ILF, heart rate variability-based (HRV) biofeedback, classical rewarding of fixed EEG bandwidths and QEEG-based NFB, would be equally effective in reduction of post-COVID complications. Since all of these aforementioned NFB modalities were showed to be effective at improving of symptoms that overlap with post-COVID complications, such as sleep problems, anxiety, fatigue, depression and pain [86], it might be worth-investigating to compare effect of different NFB modalities on post-COVID complications. We believe that future longitudinal and cross-sectional studies should be done to clarify this research question.

## 5 Limitations

We feel there are some general limitations that need to be mentioned. First, we used small sample of the participants. Furthermore, there was quite a wide age-range of participant which also might have caused some unsystematic variations to the results. In addition, there might have been some information noise caused by recruitment of the participants based on their subjective self-reference of post-COVID complications. Secondly, we did not include control group, so it is hard to differentiate between influences of true effects of NFB and other factors. Third, the exact aetiology of post-COVID manifestations is unknown due to the absence of entering medical examination. There might have been also some noise in interpretation of our results by discussing other studies investigating effects of brain non-invasive stimulation as they differed in factors such as number of intervention sessions, modalities of non-invasive stimulation, and types of questionnaires, study protocols, and target non-clinical or clinical populations. Last but not least, the absence of use of objective measurement of neurophysiological activity limits our ability to determine the relation between neural correlates of post-COVID complications and NFB-related processes.

## 6 Conclusion

In this open-label pilot study, we investigated the effect of 5 NFB sessions exploiting Othmer method of individual IOF on post-COVID fatigue, anxiety and depression. We are coming with the following findings: According to Beck Depression Inventory (2. version) and Beck Anxiety Inventory, NFB significantly reduced anxiety and depression. There was found positive correlation between depression and fatigue, anxiety and fatigue, and between depression and anxiety. There was observed inter-correlated improvement of fatigue and depression. In discussion, we focused on interpretation of our findings. According to these findings, we propose NFB is feasible for treatment of post-COVID complications as well as for studying their inter-relatedness.

In spite of aforementioned limitations, we feel that the findings of our pilot study are relevant and worth-studying and we hope that they will be useful for the future research. Future randomized controlled trials with robust sample would be necessary to investigate feasibility of neurofeedback therapy for COVID-19 complications.

## Supporting information

S1 Protocol Projekt „Efekt EEG biofeedbacku na neurologické post-COVID symptomy”(original Czech version). (DOCX)

S1 File Minimal dataset. (DOCX)

S1 Checklist TREND statement checklist. (DOCX)
