# Supplementary material for: Effect of neurofeedback therapy on neurological post-COVID-19 complications (A pilot study)
Source: PLoS One. 2022 Jul 27;17(7):e0271350. doi: 10.1371/journal.pone.0271350 (PMC9328527; doi:10.1371/journal.pone.0271350)
Supplement: S1 Protocol — (DOCX) [file pone.0271350.s001.docx]

**Projekt „Efekt EEG biofeedbacku na neurologické post-COVID symptomy“ (Original Czech version)**

**Anotace**

Neurofeedback (NFB) představuje neinvazivní, bezpeční a snadno aplikovatelnou techniku schopnou modulovat neuronální aktivitu. Jedná se o sebe-učení mozku pomocí tzv. biologické zpětné vazby. Když mozek dostane okamžitou, cílenou a přesnou informaci o svém aktuálním stavu, může se váš mozek naučit, jak se na základě zpětné vazby dostat do optimálního stavu, v kterém může správně plnit svoje funkce. Elektroencefalogram (EEG) biofeedback-využívá EEG signál ze skalpových elektrod umístněných na cílových mozkových lokalitách trénované osoby. EEG biofeedback se v terapeutické i experimentální praxi používá ke zlepšení různých mozkových funkcí jako kognitivních funkcí, spánku, motorických dovedností a řadu jiných. EEG biofeedback byl řadou studií prokázán jako účinná terapeutická metoda ke zlepšení neurologických symptomů jako únava, migrény, nespavost, závraty, záchvaty, úzkost a deprese. Interhemisferální trénink temporálních oblastí (T3-T4) s individuální optimální odměňovací frekvencí představuje tzv. Othmerovou NFB metodu, která byla prokázaná jako úspěšná u redukcí neurologických potíží jako migrény, záchvaty, závraty, prudké změny nálady a neschopnost udržení spánku. Posilování amplitudy individuální optimální NFB frekvence mezi 2 elektrody je doprovázená stavem optimální bdělosti a uvolněnosti, tzv. stavem optimálního nabuzení (arousal). Příliš nízka NFB frekvence je doprovázená stavem únavy, apatie, smutku. Naopak, příliš vysoká tréninková NFB frekvence vede k tzv. high arousal, který se projeví jako úzkost, nekontrolovatelný tok myšlenek, bušení srdce, noční mory. Předpokladáme, že nalezení a a následujících 5 NFB sezení odměňování optimální NFB frekvence povede k redukci post-COVIDové úpravy. Dál předpokládáme, že interhemisferální NFB trénink povede ke zlepšení post-COVIDových komplikací jako závraty, migrény, prudké výkyvy nálady, záchvaty a neschopnost udržení spánku. Pro zahrnutí do této studie bude potřeba přítomnost alespoň 1 z těchto symptomů, které nebyly přítomné před onemocněním COVID-19: jako únava, závraty, migrény, prudké výkyvy nálady, záchvaty a neschopnost udržení spánku Protože se tyto neurologické symptomy vyskytují u tzv. neurologického post-COVID syndromu, cílem této studie bude ověřit, jestli interhemisferální EEG biofeedback může představovat účinnou terapeutickou metodu pro zlepšení post-COVID neurologických komplikací.

**Cíl**

Cílem je ověření hypotézy, že 5 EEG biofeedback sezení signifikantně redukuje závažnost neurologických post-COVID symptomů, které budou pomocí standardizovaných dotazníků testované před a po neurofeedback intervencí.

**Metodika**

Experiment bude realizován na dobrovolnících starších jako 18 let s positivní anamnézou prodělaného onemocnění COVID-19 (potvrzeného na základě předložení positivního výsledku testu reverse-transcriptase polymerase chain reaction (PCR), kteří podepíší informovaný souhlas. Pro zahrnutí do studie od posledního positivního výsledku PCR testu nesmí uplynout míň jako 21 dní a zároveň po dobu alespoň posledních 14 dní nesmí být přítomné symptomy akutního onemocnění jako (sub)febrílie, dušnost, myalgie. Během první experimentální návštěvy, dobrovolník absolvuje vstupní anamnestický rozhovor ohledně svého zdravotního stavu a vyplní standardizované dotazníky určené pro měření míru závažnosti těchto neurologických symptomů: únava, migrény, nespavost, závraty, záchvaty, úzkost a deprese. V druhé části prvního sezení proběhne první NFB trénink během kterého bude probíhat nalezení individuální optimální tréninkové frekvence. Pak budou následovat další 4 NFB sezení. Délka 1 NFB tréninku bude 30-45 min, což je považováno za fyziologicky bezpečnou časovou délku splňující mezinárodní standardy neurofeedback metody. NFB trénink může být zkrácen na individuální žádost trénujícího, nebo pokud u trénujícího dojde například k únavě. Před, během a po každém NFB sezení bude experimentátor zjišťovat subjektivní reakce dobrovolníka na trénink a na základě jeho subjektivních reakcí se bude NFB odměňovaná frekvence a délka NFB trénink přizpůsobovat a optimalizovat vzhledem ke stavu dobrovolníka studie. Po absolvování všech 5 NFB sezení, bude následovat další amnestický rozhovor a vyplnění standardizovaných dotazníků určené pro měření míry závažnosti těchto neurologických symptomů: únava, migrény, nespavost, závraty, záchvaty, úzkost a deprese. NFB trénink bude provádět osoba (Mária Orendáčová), která má na to absolvovaný certifikovaný kurz, ´´Biofeedback pro profesionály´´, absolvovaný v Biofeedback Institut v Praze, kde dotyčná osoba rok a půl, po absolvování biofeedback kurzu, pracovala v NFB klinické praxi.

**Časový plán**

Sběr dat: 15.8.2021 - 30.01.2022

Zpracování dat: 30.01.2022 - 30.02.2022

Vyhodnocení dat: 30.02.2022 - 15.03.2022

**Projektový tým**

Mgr. Mária Orendáčová, maria.orendacova@lf3.cuni.cz

RNDr. Eugen Kvašňák, PhD., eugen.kvasnak@lf3.cuni.cz

**Project „Effect of EEG biofeedback on neurological post-COVID symptoms (English translation of the original)**

**Annotation**

Neurofeedback (NFB) represents non-invasive, safe and feasible method for modulation of neural activity. It is based on self-regulation of brain by means of providing of biological feedback. Provided that brain receives immediate, targeted and exact information about its actual state, due to the presence of biological feedback, the brain can learn to tune itself into its optimal state in which it can work properly. Electroencephalogram (EEG) biofeedback senses EEG signal from the scalp electrodes which are placed on the participants' head. In therapeutic and clinical practice, EEG biofeedback has been used for improving a variety of brain functions such as cognitive functions, sleep, motor functions and many others. EEG biofeedback has been proven to represent effective therapeutic strategy for improvement of neurological symptoms such as fatigue, migraines, insomnia, seizures, dizziness, anxiety and depression. Interhemispheric NFB training of temporal areas exploiting individual optimal reward EEG frequency represents so-called Othmer NFB method, which was proven to be effective in the reduction of migraines, seizures, abrupt mood swings and maintenance insomnia. Reinforcement of amplitude of individual optimal NFB frequencies between 2 electrodes is accompanied with the state of optimal alertness and relaxation which represent so-called optimal arousal. Too low NFB-rewarded EEG frequency is accompanied with subjective feelings of fatigue, apathy and sadness. On the other hand, too high NFB-rewarded EEG frequency results in the states such as anxiety, uncontrolled stream of thoughts, tachycardia and nightmares. Our hypothesis is that finding optimal NFB-rewarded EEG frequency and 5 NFB sessions will reduce level of post-COVID complications such as dizziness, migraines, mood swings, seizures, fatigue, anxiet , depression and maintenance insomnia. In order to be included in this study, the presence of at least one symptom of the following symptoms will be required which was/were not present prior to COVID-19: dizziness, migraines, mood swings, seizures, fatigue, anxiety, depression and maintenance insomnia. Because such symptoms occur in so-called neurological post-COVID syndrome, the aim of this study will be to investigate whether interhemispheric EEG biofeedback might represent efficient method for improvement of post-COVID complications.

**Aim of study:**

The aim of this study will be to verify hypothesis that 5 NFB studies will significantly reduce severity of neurological post-COVID symptoms which will be measured before and after NFB intervention.

**Methodology:**

The experimental procedure will be done on volunteers older than 18 years having positive anamnesis of COVID-19 (confirmed by reverse-transcriptase polymerase chain reaction (PCR) ) who will sign informed consent. To be included in the study, there cannot be less than 21 days from the last positive PCR test and at the same time, in the period of the last 14 days, there cannot be present any symptoms indicating the presence of acute symptoms such as (sub) febrile state, dyspnoea and myalgia. During the first experimental session, the volunteering participants will complete entering anamnestic interview regarding his/her health condition and fill standardized questionnaires used for measuring the severity of the following neurological symptoms: fatigue, migraines, insomnia, dizziness, seizures, anxiety and depression. In the second part of the first experimental session, there will be NFB part during which individual optimal NFB-rewarded EEG frequency will be found. Then, 4 more sessions will be given. Period of one NFB session will fall within 30-45 minutes which is considered to be physiologically safe and it meets international standard for neurofeedback method. The period of NFB may be shorten due to individual request of the participant and/or provided the participants start to feel subjectively fatigued. Before, during and after each NFB sessions, the experimenter will ask participants regarding their subjective responses to NFB training and based on their subjective responses, NFB-rewarded EEG frequency or period of NFB session will be adjusted. After completing all 5 NFB sessions, there will be interview with the participants done as well as filling standardized questionnaires used for measuring severity of the following neurological symptoms: fatigue, migraines, insomnia, dizziness, seizures, anxiety and depression. NFB training will be provided by certified NFB therapist (Mária Orendáčová) who had certified course ´´Biofeedback pro profesionály´´, taken in EEG Biofeedback Institute in Prague in which the particular person worked in NFB clinical practice for 1.5 years.

**Time schedule**

Data collection: 15.8.2021 - 30.01.2022

Data analysis: 30.01.2022 - 30.02.2022

Data evaluation: 30.02.2022 - 15.03.2022

**Project team:**

Mgr. Mária Orendáčová, maria.orendacova@lf3.cuni.cz

RNDr. Eugen Kvašňák, PhD., eugen.kvasnak@lf3.cuni.cz
