# Supplementary material for: Effect of neurofeedback therapy on neurological post-COVID-19 complications (A pilot study)
Source: PLoS One. 2022 Jul 27;17(7):e0271350. doi: 10.1371/journal.pone.0271350 (PMC9328527; doi:10.1371/journal.pone.0271350)
Supplement: S1 File — (DOCX) [file pone.0271350.s002.docx]

**S2 Effect of neurofeedback on neurological post-COVID symptoms**

**Table 1 Descriptive Statistics**

| **Measured value** | **Number (N) of analyzed participants** | **Mean** | **Minimum** | **Maximum** | **Standard deviation** | **Confidence interval** |
| --- | --- | --- | --- | --- | --- | --- |
| **Fatigue Assessment Scale Score before NFB** | N=9 | 32.77778 | 22 | 45 | 9.06612 | 95% |
| **Fatigue Assessment Scale Score immediately after NFB** | N=9 | 25.88889 | 19 | 37 | 6.88194 | 95% |
| **Fatigue Assessment Scale Score one week after NFB** | N=9 | 26.66667 | 17 | 42 | 8.42615 | 95% |
| **Fatigue Assessment Scale Score one month after NFB** | N=9 | 26.00000 | 18 | 39 | 8.20061 | 95% |
| **Beck Anxiety Inventory Score before NFB** | N=8 | 32.50000 | 25 | 49 | 8.94427 | 95% |
| **Beck Anxiety Inventory Score immediately after NFB** | / | / | / | / | / | / |
| **Beck Anxiety Inventory Score one week after NFB** | N=8 | 19.75000 | 3 | 36 | 10.53904 | 95% |
| **Beck Anxiety Inventory Score one month after NFB** | N=8 | 18.50000 | 6 | 33 | 9.28901 | 95% |
| **Beck Depression Inventory Score (second version) before NFB** | N=6 | 26.66667 | 17 | 42 | 9.89276 | 95% |
| **Beck Depression Inventory Score (second version) immediately after NFB** | N=6 | 14.50000 | 3 | 20 | 6.92098 | 95% |
| **Beck Depression Inventory Score (second version) one week after NFB** | N=6 | 14.50000 | 3 | 20 | 5.78792 | 95% |
| **Beck Depression Inventory Score (second version) one month after NFB** | N=6 | 15.33330 | 4 | 27 | 9.60555 | 95% |

**Table 1 Descriptive Statistics.** This table shows values for descriptive statistics such as number (N) of analyzed participants for the particular post-COVID symptoms, mean, minimum, maximum, standard deviations and confidence interval value. Measured values (bold text in the first column in the left) include score for Fatigue Assessment Scale, Beck Anxiety Inventory, Beck Depression Inventory (second version) before, immediately after, one week after and one month after NFB. The symbol ,,/,, in the line ,, Beck Anxiety Inventory Score immediately after NFB,, indicates no measurement was done because of specificity of the particular questionnaire.

**Table 2 Score for Fatigue Assessment Scale**

| **ID of participant** | **Before NFB** | **Immediately after NFB** | **1 week after NFB** | **1 month after NFB** |
| --- | --- | --- | --- | --- |
| **1** | 26 | 21 | 17 | 20 |
| **2** | 42 | 37 | 34 | 33 |
| **3** | 29 | 21 | 22 | 21 |
| **4** | 24 | 25 | 23 | 18 |
| **5** | 45 | 37 | 42 | 39 |
| **6** | 44 | 26 | 35 | 37 |
| **7** | / | / | / | / |
| **8** | 36 | 20 | 22 | 19 |
| **9** | 22 | 19 | 19 | 21 |
| **10** | 27 | 27 | 26 | 26 |
|  |  |  |  |  |

**Table 2 Score for Fatigue Assessment Scale.** In this table, score of Fatigue Assessment Scale for individual participants is recorded. Data were collected before NFB, immediately after NFB, 1 week after NFB and 1 month after NFB (Columns with bold-text title). Abbreviation ,,ID,, stands for identification number of the individual participants. The presence of ,,/,, for Participant 7 means the participant did not meet inclusion criteria for having at least 22 points indicating ,,no presence of fatigue,, and therefore the participant/s is/are not included in analysis of group of participants in which the presence of fatigue was classified to occur)

**Table 3 Score for Beck Anxiety Inventory**

| **ID of participant** | **Before NFB** | **Immediately after NFB** | **1 week after NFB** | **1 month after NFB** |
| --- | --- | --- | --- | --- |
| **1** | 28 | / | 3 | 17 |
| **2** | 35 | / | 22 | 28 |
| **3** | / | / | / | / |
| **4** | 25 | / | 21 | 20 |
| **5** | 31 | / | 27 | 22 |
| **6** | 42 | / | 36 | 33 |
| **7** | 25 | / | 7 | 6 |
| **8** | 25 | / | 19 | 8 |
| **9** | / | / | / | / |
| **10** | 49 | / | 23 | 14 |

**Table 3 Score for Beck Anxiety Inventory .** In this table, score of Beck Anxiety Inventory for individual participants is recorded. Data were collected before NFB, immediately after NFB, 1 week after NFB and 1 month after NFB (Columns with bold-text title). Abbreviation ,,ID,, stands for identification number of the individual participants. The presence of ,,/,, for Participants 3 and 9 means they did not meet inclusion criteria for having at least moderate level of anxiety, therefore they are not analyzed for group of participants having depression). ,,/,, in column ,,Immediately after NFB,, means that score for Beck Anxiety Inventory for all participants is not evaluated immediately after NFB, due to specific parametres of that questionnaire.

**Table 4 Score for Beck Depression Inventory (Second version)**

| **ID of participant** | **Before NFB** | **Immediately after NFB** | **1 week after NFB** | **1 month after NFB** |
| --- | --- | --- | --- | --- |
| **1** | / | / | / | / |
| **2** | 33 | 19 | 20 | 26 |
| **3** | / | / | / | / |
| **4** | / | / | / | / |
| **5** | 22 | 20 | 18 | 17 |
| **6** | 42 | 19 | 20 | 27 |
| **7** | 17 | 3 | 6 | 4 |
| **8** | 29 | 17 | 10 | 9 |
| **9** | / | / | / | / |
| **10** | 17 | 9 | 13 | 9 |
|  |  |  |  |  |

**Table 4 Score for Beck Depression Inventory (Second version).** In this table, score of Beck Depression Inventory (Second version) for individual participants is recorded. Data were collected before NFB, immediately after NFB, 1 week after NFB and 1 month after NFB (Columns with bold-text title). Abbreviation ,,ID,, stands for identification number of the individual participants. The presence of ,,/,, for Participants 1,3, 4 and 9 means they did not meet inclusion criteria for having at least borderline level of depression, therefore they are not analyzed for group of participants having depression)

**Table 5 Correlations between Fatigue, anxiety, depression**

| ID | **Before NFB** | Fatigue | Anxiety | Depresssion | **Imm.after NFB** | Fatigue | Anxiety | Depression | **1 week after NFB** | Fatigue | Anxiety | Depression | **1 month after NFB** | Fatigue | Anxiety | Depression |
| --- | --- | --- | --- | --- | --- | --- | --- | --- | --- | --- | --- | --- | --- | --- | --- | --- |
| 1 |  | 26 | 28 | 13 |  | 21 | / | 9 |  | 17 | 3 | 1 |  | 20 | 17 | 9 |
| 2 |  | 42 | 35 | 33 |  | 37 | / | 19 |  | 34 | 22 | 26 |  | 33 | 28 | 26 |
| 3 |  | 29 | 15 | 14 |  | 21 | / | 7 |  | 22 | 11 | 5 |  | 21 | 9 | 7 |
| 4 |  | 24 | 25 | 8 |  | 25 | / | 6 |  | 23 | 21 | 3 |  | 18 | 20 | 2 |
| 5 |  | 45 | 31 | 22 |  | 37 | / | 20 |  | 42 | 27 | 18 |  | 39 | 22 | 17 |
| 6 |  | 66 | 42 | 42 |  | 44 | / | 19 |  | 35 | 36 | 20 |  | 37 | 33 | 27 |
| 7 |  | 15 | 25 | 17 |  | 11 | / | 3 |  | 12 | 7 | 6 |  | 11 | 6 | 4 |
| 8 |  | 36 | 25 | 29 |  | 20 | / | 17 |  | 22 | 19 | 10 |  | 19 | 8 | 9 |
| 9 |  | 22 | 15 | 14 |  | 19 | / | 9 |  | 19 | 11 | 10 |  | 21 | 14 | 11 |
| 10 |  | 27 | 49 | 17 |  | 27 | / | 9 |  | 26 | 23 | 13 |  | 26 | 14 | 9 |

**Table 5 Correlations between fatigue, anxiety, depression**-Based on data from Fatigue Assessment Scale, Beck Anxiety Inventory and Beck Depression Inventory, correlation analysis was done between means for score of fatigue,anxiety and depression before NFB, immediately (imm.) after NFB, one week after NFB and one month after NFB (4 columns with title-bold text). Abbreviation ,,ID,, stands for identification number of the particular participant. In column titled as ,, Fatigue,, score of the individual participants for Fatigue Assessment Scale is recorded. In column titled as ,, Depression,, score of the individual participants for Beck Depression Inventory (Second version) is recorded. In column titled as ,, Anxiety,, score of the individual participants for Beck Anxiety Inventory is recorded. In this correlation analysis, all scores for fatigue, depression and anxiety for all 10 participants are analyzed regardless their baseline score (before NFB intervention). Score for Beck anxiety inventory is not analyzed immediately after NFB due to specifity of parametres for that questionnaire.

**Figures**


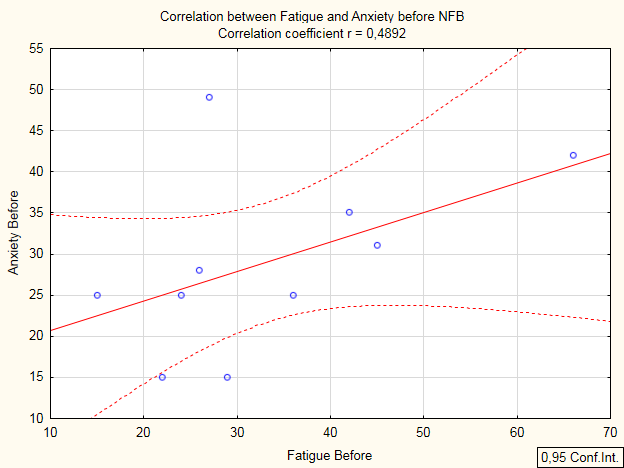
 Figure 1 Correlation between fatigue and anxiety before NFB. This figure shows correlation coefficient (r) between fatigue and anxiety before NFB. Axis X shows the score for fatigue before NFB (Fatigue Before) and Axis Y shows the score for anxiety (Anxiety Before). Confidence interval (Conf.Int.) is 0.95.


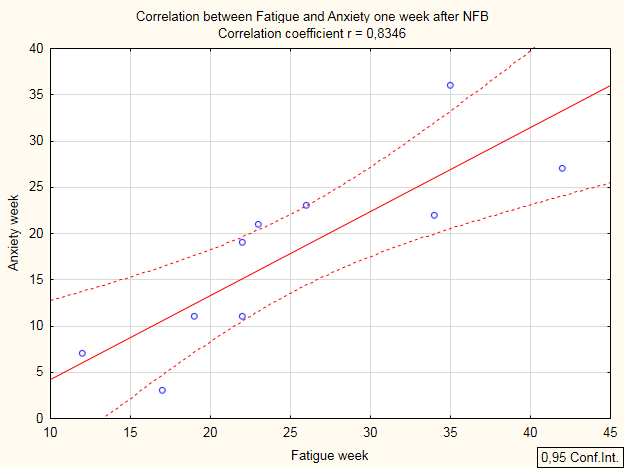
 Figure 2 Correlation between fatigue and anxiety one week after NFB. This figure shows correlation coefficient (r) between fatigue and anxiety one week after NFB. Axis X shows the score for fatigue one week after NFB (Fatigue week) and Axis Y shows the score for anxiety (Anxiety week). Confidence interval (Conf.Int.) is 0.95.


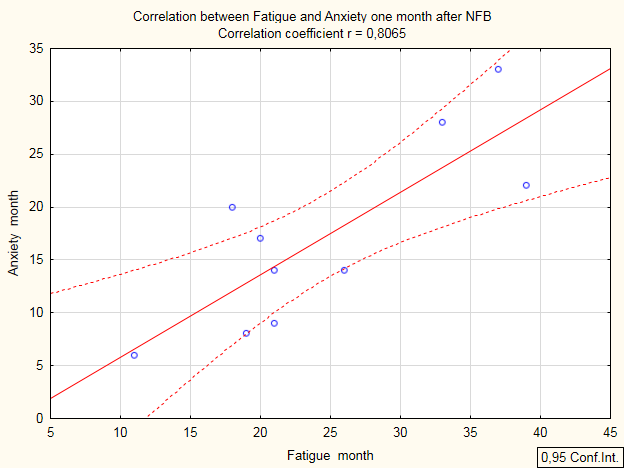
 Figure 3 Correlation between fatigue and anxiety one month after NFB. This figure shows correlation coefficient (r) between fatigue and anxiety one month after NFB. Axis X shows the score for fatigue one month after NFB (Fatigue month) and Axis Y shows the score for anxiety (Anxiety month). Confidence interval (Conf.Int.) is 0.95.


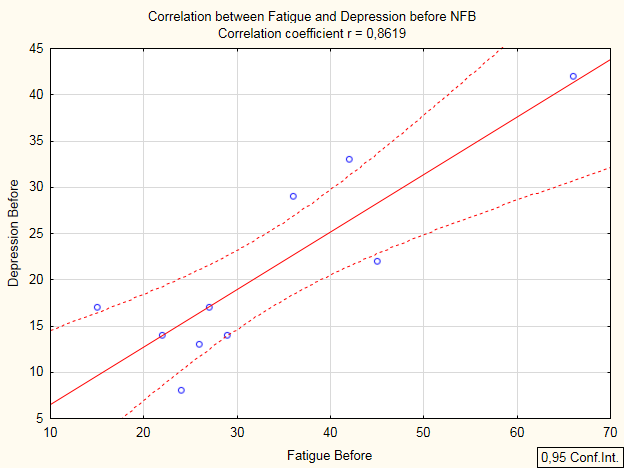
 Figure 4 Correlation between fatigue and anxiety before NFB. This figure shows correlation coefficient (r) between fatigue and depression before NFB. Axis X shows the score for fatigue before NFB (Fatigue Before) and Axis Y shows the score for depression (Depression Before). Confidence interval (Conf.Int.) is 0.95.


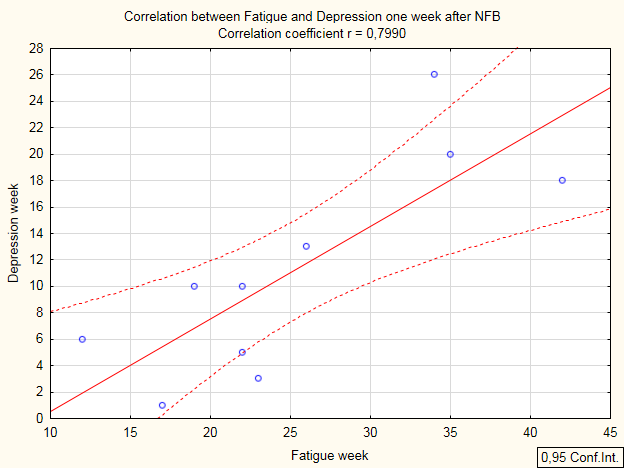
 Figure 6 Correlation between fatigue and anxiety one week after NFB. This figure shows correlation coefficient (r) between fatigue and depression one week after NFB. Axis X shows the score for fatigue one week after NFB (Fatigue week) and Axis Y shows the score for depression (Depression week). Confidence interval (Conf.Int.) is 0.95.


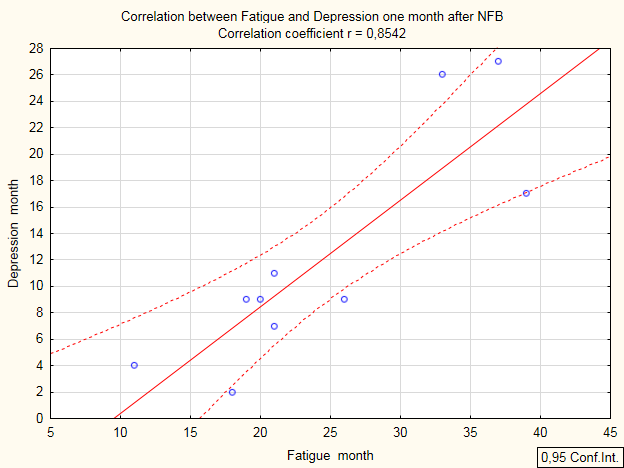
 Figure 7 Correlation between fatigue and anxiety one month after NFB. This figure shows correlation coefficient (r) between fatigue and depression one month after NFB. Axis X shows the score for fatigue one month after NFB (Fatigue month) and Axis Y shows the score for depression (Depression month). Confidence interval (Conf.Int.) is 0.95.


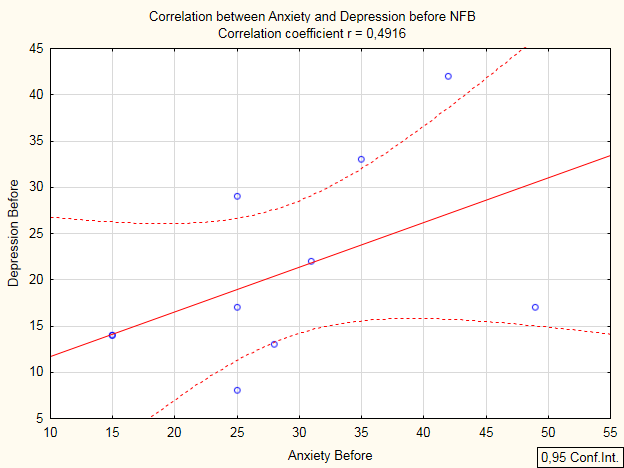


Figure 8 Correlation between anxiety and depression before NFB. This figure shows correlation coefficient (r) between anxiety and depression before NFB. Axis X shows the score for anxiety before NFB (Anxiety Before) and Axis Y shows the score for depression (Depression Before). Confidence interval (Conf.Int.) is 0.95.


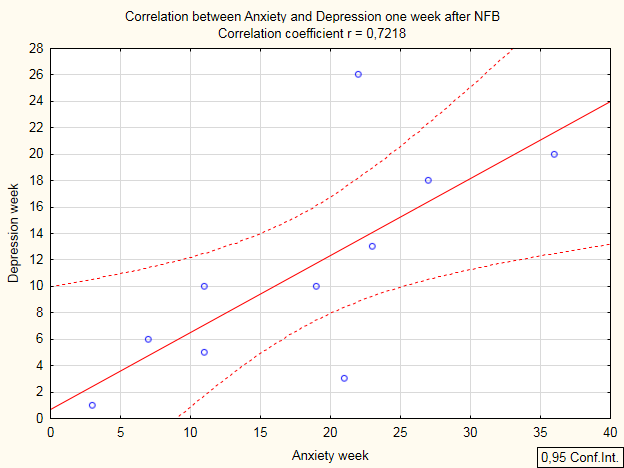
 Figure 9 Correlation between anxiety and depression one week after NFB. This figure shows correlation coefficient (r) between anxiety and depression one week after NFB. Axis X shows the score for anxiety one week after NFB (Anxiety week) and Axis Y shows the score for depression (Depression week). Confidence interval (Conf.Int.) is 0.95.


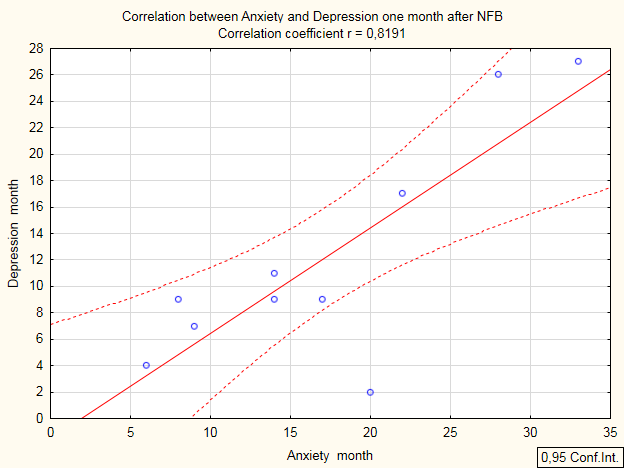
 Figure 10 Correlation between anxiety and depression one month after NFB. This figure shows correlation coefficient (r) between anxiety and depression one month after NFB. Axis X shows the score for anxiety one month after NFB (Anxiety month) and Axis Y shows the score for depression (Depression month). Confidence interval (Conf.Int.) is 0.95.


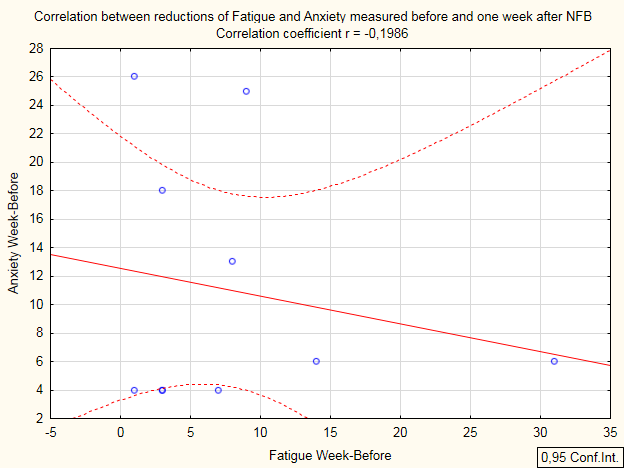
 Figure 11 Correlation between reductions in fatigue and anxiety before and one week after NFB. This figure shows correlation coefficient (r) between reductions in fatigue and anxiety (before NFB vs. one week after NFB. Axis X shows the difference in score for fatigue between before NFB and one week after NFB (Fatigue Week-Before) and Axis Y shows the difference in score for anxiety between before NFB and one week after NFB (Anxiety Week-Before). Confidence interval (Conf.Int.) is 0.95.


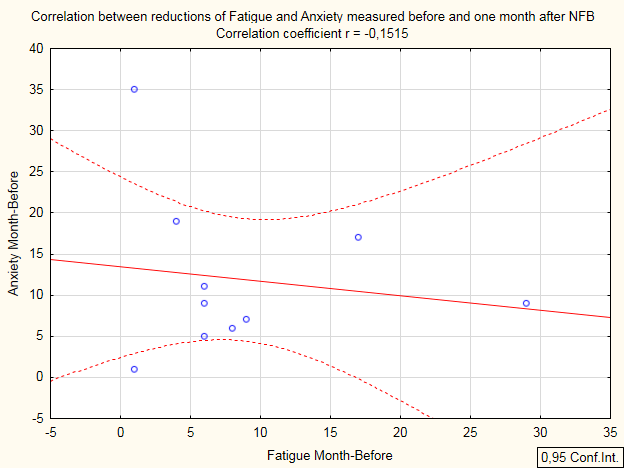
 Figure 12 Correlation between reductions in fatigue and anxiety before and one month after NFB. This figure shows correlation coefficient (r) between reductions in fatigue and anxiety (before NFB vs. one month after NFB. Axis X shows the difference in score for fatigue between before NFB and one month after NFB (Fatigue Month-Before) and Axis Y shows the difference in score for anxiety between before NFB and one month after NFB (Anxiety Month-Before). Confidence interval (Conf.Int.) is 0.95.


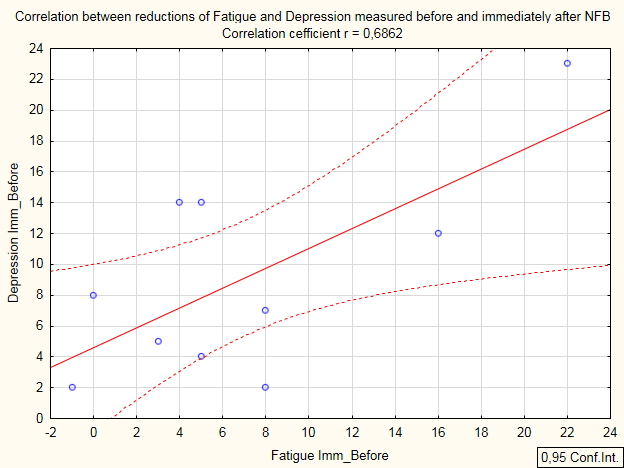
 Figure 13 Correlation between reductions in fatigue and depression before and immediately after NFB. This figure shows correlation coefficient (r) between reductions in fatigue and depression (before NFB vs. immediately after NFB. Axis X shows the difference in score for fatigue between before NFB and immediately after NFB (Fatigue Imm-Before) and Axis Y shows the difference in score for depression between before NFB and immediately after NFB (Depression Imm-Before). Confidence interval (Conf.Int.) is 0.95.


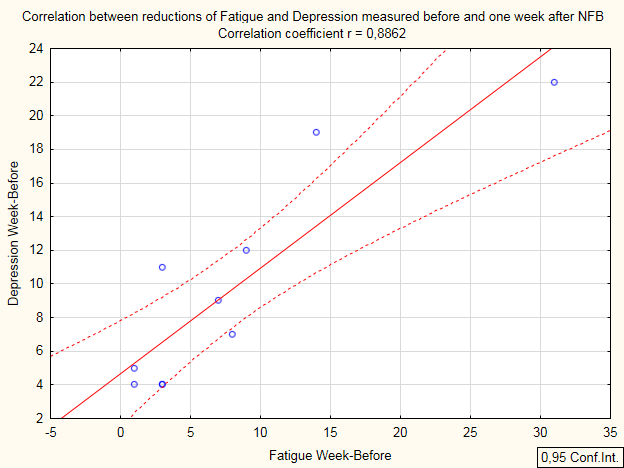
 Figure 14 Correlation between reductions in fatigue and depression before and one week after NFB. This figure shows correlation coefficient (r) between reductions in fatigue and depression (before NFB vs. one week after NFB. Axis X shows the difference in score for fatigue between before NFB and immediately after NFB (Fatigue Week-Before) and Axis Y shows the difference in score for depression between before NFB and immediately after NFB (Depression Week-Before). Confidence interval (Conf.Int.) is 0.95.


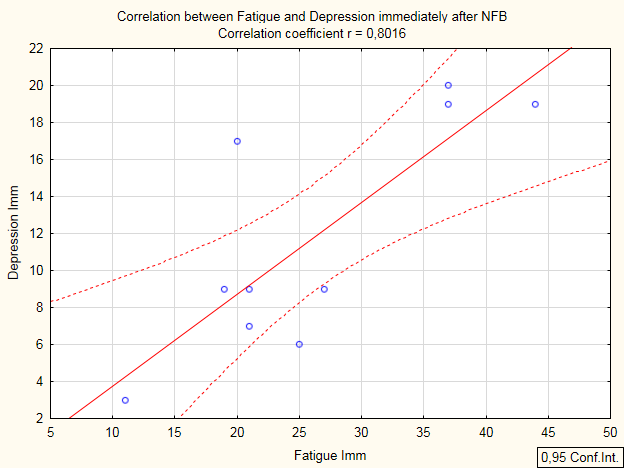
 Figure 15 Correlation between fatigue and anxiety immediately after NFB. This figure shows correlation coefficient (r) between fatigue and depression immediately after NFB. Axis X shows the score for fatigue immediately after NFB (Fatigue Imm.) and Axis Y shows the score for depression (Depression Imm.). Confidence interval (Conf.Int.) is 0.95.


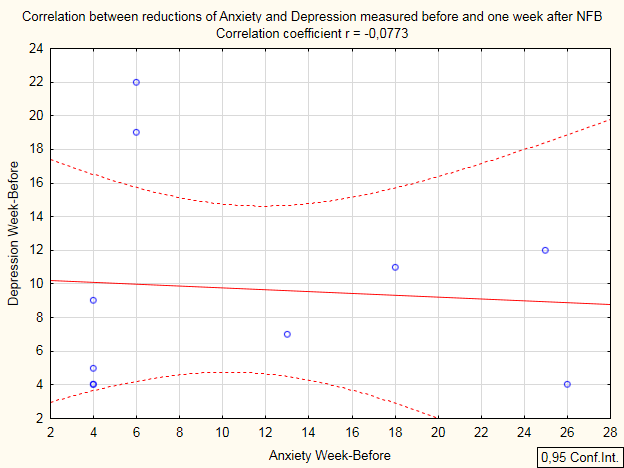
 Figure 16 Correlation between reductions in anxiety and depression before and one week after NFB. This figure shows correlation coefficient (r) between reductions in anxiety and depression (before NFB vs. one week after NFB. Axis X shows the difference in score for anxiety between before NFB and one week after NFB (Anxiety Week-Before) and Axis Y shows the difference in score for depression between before NFB and one week after NFB (Depression Week-Before). Confidence interval (Conf.Int.) is 0.95.


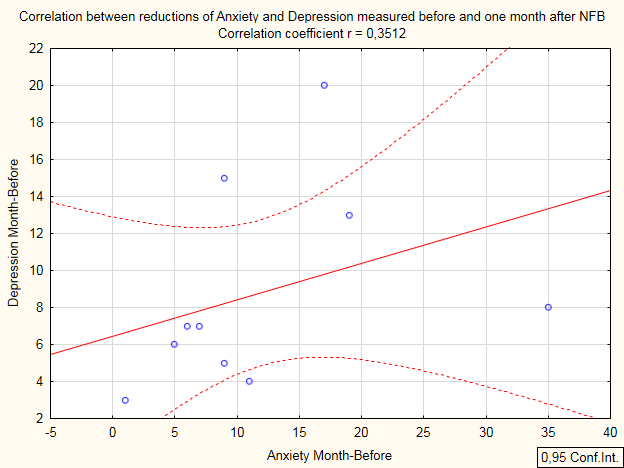
 **Figure 17 Correlation between reductions in anxiety and depression before and one month after NFB**. This figure shows correlation coefficient (r) between reductions in anxiety and depression (before NFB vs. one month after NFB. Axis X shows the difference in score for anxiety between before NFB and one month after NFB (Anxiety Month-Before) and Axis Y shows the difference in score for depression between before NFB and one month after NFB (Depression Month-Before). Confidence interval (Conf.Int.) is 0.95.
